# The Gradience of Multilingualism in Typical and Impaired Language Development: Positioning Bilectalism within Comparative Bilingualism

**DOI:** 10.3389/fpsyg.2016.00037

**Published:** 2016-02-10

**Authors:** Kleanthes K. Grohmann, Maria Kambanaros

**Affiliations:** ^1^Department of English Studies, University of CyprusNicosia, Cyprus; ^2^Cyprus Acquisition TeamNicosia, Cyprus; ^3^Department of Rehabilitation Sciences, Cyprus University of TechnologyLimassol, Cyprus

**Keywords:** biolinguistics, clitics, comparative linguality, dialect, executive control, Greek, specific language impairment, socio-syntax

## Abstract

A multitude of factors characterizes bi- and multilingual compared to monolingual language acquisition. Two of the most prominent viewpoints have recently been put in perspective and enriched by a third (Tsimpli, 2014): age of onset of children's exposure to their native languages, the role of the input they receive, and the timing in monolingual first language development of the phenomena examined in bi- and multilingual children's performance. This article picks up a fourth potential factor (Grohmann, 2014b): language proximity, that is, the closeness between the two or more grammars a multilingual child acquires. It is a first attempt to flesh out the proposed gradient scale of multilingualism within the approach dubbed “comparative bilingualism.” The empirical part of this project comes from three types of research: (i) the acquisition and subsequent development of pronominal object clitic placement in two closely related varieties of Greek by bilectal, binational, bilingual, and multilingual children; (ii) the performance on executive control tasks by monolingual, bilectal, and bi- or multilingual children; and (iii) the role of comparative bilingualism in children with a developmental language impairment for both the diagnosis and subsequent treatment as well as the possible avoidance or weakening of how language impairment presents.

## Introduction

Language acquisition in the multicultural, multiethnic, and especially multilingual environments in which children grow up more and more frequently needs to be paid, correspondingly, closer attention to. This much needed attention concerns a range of educational and sociological issues, just as it is relevant for all matters related to language assessment: determining milestones in typically developing children's language development, assessing problems with language growth early on, diagnosing language impairment, and subsequently developing appropriate speech–language therapy and intervention. Beyond these practical needs, there is also a larger research interest in multilingual acquisition that allows a better view into the underlying cognitive structures.

From the earliest studies of language development, it has become very clear that monolingual language acquisition differs greatly from bi- and multilingual language acquisition—despite fundamental similarities. Depending on where one sets the boundaries, it might even be held that monolingualism does not really exist, *sensu stricto* (think of different sociolects, idiolects, and so on that every speaker commands). This said, the multilingual child faces a number of obstacles that do not factor into monolingual mother tongue acquisition. Two obvious and well studied factors are the age of onset of children's exposure to each of their two or more native languages and the role, in terms of quantity and quality, of the input they receive in each (e.g., Meisel, 2009; Genesee et al., 2011; Unsworth et al., 2014). In addition, Tsimpli (2014) suggests that the timing in monolingual first language development of the phenomena examined in bi- and multilingual children's performance influences whether a particular linguistic phenomenon is acquired (very) early or late. One aspect explored in the present article is a potential fourth factor (Grohmann, 2014b): language proximity, that is, the closeness between the two or more grammars a multilingual child acquires.

Since this article reports research carried out in Cyprus with local acquirers, we will set the scene by briefly laying out the notion of language proximity as relevant for the context of Greek-speaking Cyprus. In the following, we aim to flesh out the proposed gradient scale of multilingualism within the approach dubbed “comparative bilingualism.” The empirical part of this research comes from three types of research. We first report data collected on the acquisition and subsequent development of object clitic placement in the two varieties of Greek spoken in Cyprus by bilectal, binational, bilingual, and multilingual children. The second study draws from the performance on executive control tasks by monolingual, bilectal, and bi- or multilingual children. And finally we address a third line of inquiry on the issue of comparative bilingualism, vis-à-vis multilingual language acquisition: the role of bilingualism in children with developmental language impairment, where we will also briefly consider the diagnosis and subsequent treatment of multilingual (language-)developmentally impaired children. Couched within a biolinguistic outlook to language growth, the research agenda sketched here will eventually offer the opportunity to study the neurobiology of language in different (multi)lingual individuals at different ages. This will be reflected in the Discussion and Outlook, which returns full circle to the idea of “comparative bilingualism” by first extending it further (qua a gradient scale of *multi*lingualism), then connecting it to cognitive neuroscientifically relevant research within the new research area of “comparative biolinguistics” (phenotypic variation such as different manifestations of language impairment and breakdown), and finally suggesting a more holistic agenda for future research investigations: “comparative linguality.”

## Approaching language proximity for language acquisition in cyprus

We begin by echoing Grohmann's (2014b) suggestion of a fourth factor for multilingual language development: language proximity. In fact, the present article builds on Grohmann (2014b), a brief commentary on the epistemological paper by Tsimpli (2014), filling in some details and expanding on others. With respect to proximity, considering the linguistic closeness or distance between the grammars of all languages a bi- or multilingual child acquires will then allow further entertaining the notion of *comparative bilingualism*. The larger research agenda is one in which comparable phenomena are systematically investigated across bi- and multilingual populations with different language combinations, ideally arranged according to purely structural/grammatical, language typological, or perhaps even areal proximity (e.g., a large body of research in the wake of Thomason and Kaufman, 1988). This is a much larger research project for which “language proximity” first has to be properly defined, which we will leave for future considerations; we are grateful to the reviewers for fruitful discussion and constructive feedback on this issue. It will also have to be decided whether the same measurements of proximity are relevant for bi-/multilingual first language acquisition (Barac and Bialystok, 2012) as it has been argued to apply for second language acquisition (Bialystok, 1997; Birdsong and Molis, 2001) and learning (Ringbom, 2006; Ringbom and Jarvis, 2009; Ceñoz and Gorter, 2011), third language acquisition (see Falk and Bardell, 2010, for an overview), especially beyond much studied phonological influence (Llama et al., 2009; Marx and Mehlhorn, 2010), attrition (Montrul, 2008), or, further removed from acquisition factors, for other aspects of language contact (Thomason, 2001; Aikhenvald, 2007; Jarvis and Pavlenko, 2008).

Our present contribution pursues a much more graspable goal, however, namely to compare different populations of Greek speakers on the same linguistic and non-linguistic tools. These include lexical and morphosyntactic tasks, but also measures on language proficiency, pragmatics, and especially executive control. Our populations range from monolingual children growing up in Greece to multilingual children growing up in Cyprus, with several “shades” in between, all centered around the closeness between the language of Greece (Demotic Greek, typically referred to by linguists as Standard Modern Greek) and the native variety of Greek spoken in Cyprus (Cypriot Greek, which itself comes in different flavors ranging from basi- to acrolect). Detailed family and language history background information was also collected for all participants.

The official language of Greek-speaking Cyprus is Standard Modern Greek (SMG), while the everyday language, hence the variety acquired natively by Greek Cypriots, is Cypriot Greek (CG). Calling CG a dialect of SMG as opposed to treating it as a different language is largely a political question; the proximity between the two is very high, and obviously so: The two varieties largely share a common lexicon, sound structure, morphological rule system, and syntactic grammar. According to *Ethnologue* (Lewis et al., 2015), the lexical similarity between CG and SMG lies in the range of 84–93%, which the authors present as follows (http://www.ethnologue.com/ethno_docs/ introduction.asp): “Lexical similarity can be used to evaluate the degree of genetic relationship between two languages. Percentages higher than 85% usually indicate that the two languages being compared are likely to be related dialects.” It yet remains to be seen, however, what the exact criteria for such “lexical similarity” are, and whether the conclusions drawn also extend to grammatical aspects of the linguistic varieties compared. Of immediate relevance is simply the possibility which the lower bound of this purported similarity allows, namely that, at just below the “clearer” cut-off point of 85%, it is not unambiguously evident that CG should exclusively be treated as a dialect of SMG. (We concur with a reviewer who pointed out that such measurements only indicate that CG and SMG may be dialects/varieties of Modern Greek; this much is surely undisputed).

CG and SMG also differ in each of these levels of linguistic analysis as well—and at times quite substantially so (for a recent in-depth discussion, e.g., Tsiplakou, 2014). To briefly illustrate, there are naturally numerous lexical differences, as expected in any pair of closely related varieties, such as the CG feminine-marked *korua* instead of SMG neuter *koritzi* “girl.” Phonetically, CG possesses palato-alveolar consonants, in contrast to SMG, so SMG [cε′ɾɔs] becomes CG [t∫ε′ɾɔs] for *keros* “weather.” The two varieties use a different morpheme to mark 3rd person plural in present and past tenses, such as CG *pezusin* and *epezasin* instead of SMG *pezun* “they play” and *epezan* “they were playing.” On the syntactic level, SMG expresses focus by fronting to the clausal left periphery, while CG employs a cleft-like structure, which it also extensively uses in the formation of *wh*-questions. And there are even pragmatic differences such as in politeness strategies: The extensive use of diminutives in SMG is considered exaggerated by CG speakers. See, among many others, Muller (2002), Grohmann et al. (2006), Terkourafi (2007), Grohmann (2009), Arvaniti (2010), and Tsiplakou (2014) for recent discussions and further references.

Traditionally, Greek-speaking Cyprus is considered a language situation of diglossia between the sociolinguistic L(ow)-variety CG and the H(igh)-variety SMG (Newton, 1972 and much work since, building on Ferguson, 1959 [2003]; for recent overviews, see Arvaniti, 2010; Hadjioannou et al., 2011; Rowe and Grohmann, 2013). Moreover, while there is a clear basilect (“village Cypriot”), there are arguably further mesolects ranging all the way up to a widely assumed acrolect (“urban Cypriot”); Arvaniti (2010) labeled the latter Cypriot Standard Greek (CSG), a high version of CG which is closest to SMG among all CG lects. In fact, such CSG may be the real H-variety on the island, on the assumption that without native acquirers of SMG proper, the only Demotic Greek-like variety that could be taught in schools is a “Cyprified Greek,” possibly this ostensible yet elusive CSG. However, SMG can be widely heard and read in all kinds of media outlets, especially those coming from the Hellenic Republic of Greece. Note also that there is still no grammar of CSG available, no compiled list of properties, not even a term, or even existence, agreed upon; the official language is SMG.

With respect to child language acquisition, it should come as no surprise that to date no studies exist that investigate the nature, quality, and quantity of linguistic input children growing in Cyprus receive. There are simply no data available that would tell us about the proportion of basi- vs. acrolectal CG, purported CSG, and SMG in a young child's life, and whether there are differences between rural and urban upbringing or across different geographical locations. At this time, such information can only be estimated anecdotally.

We follow recent work from our research group, the Cyprus Acquisition Team (CAT), and adopt Rowe and Grohmann's (2013) term *(discrete) bilectalism* to characterize Greek Cypriot speakers in this diglossic speech community (for further discussion, see Grohmann and Leivada, 2012; Papadopoulou et al., 2014; Rowe and Grohmann, 2014), replacing our original notion of “bi-*x*” (Grohmann, 2011; Grohmann and Leivada, 2011). The first published study that addressed the role of bilectalism in language development, applied to lexical retrieval (Kambanaros and Grohmann, 2010, 2011; Kambanaros et al., 2010), is Kambanaros et al. (2013b), followed up by work comparing typically developing bilectal children to children with specific language impairment (Kambanaros et al., 2013a). To date, the lexical and morphosyntactic differences between CG and SMG qua bi-*x* or bilectalism have also featured in work on adult grammar (Grohmann and Papadopoulou, 2011) as well as specific topics in typical or impaired language, including light verb use (Grohmann and Leivada, 2013; Kambanaros and Grohmann, 2015), the comprehension and production of relative clauses (Theodorou and Grohmann, 2013), and the importance of creating an assessment tool for the diagnosis of specific language impairment for CG (Theodorou, 2013; Theodorou et al., submitted). We also raised the issue of bilectal populations as a topic of interest for research in bilingualism (e.g., Antoniou et al., 2014; Kambanaros et al., 2014), leaving the door open to classify these speakers as “bilingual” after all, once a better definition of language proximity in multilectal speakers is available beyond some notion of “second dialect (acquisition)” (cf. Siegel, 2010); this is part of our research agenda for comparative bilingualism.

With all this in place, we can assume that Greek Cypriots are typically sequential bilectal, first acquiring CG and then SMG (or something akin, such as CSG), where the onset of SMG may set in with exposure to Greek television, for example (clearly within the critical period) but most prominently with formal schooling (around first grade, possibly before, where the relation to the critical period is more blurred). What is more, due to the close relations between Cyprus and Greece (beyond language for historical, religious, political, and economic reasons), we are able to tap into two further interesting populations, all residing in Cyprus (Leivada et al., 2010): Hellenic Cypriot children, who are binational having one parent from Cyprus (Greek Cypriot) and one from Greece (Hellenic Greek), and Hellenic Greek children, with both parents hailing from Greece. Anecdotally, we could then say that binational Hellenic Cypriot children are presumably simultaneous bilectals (strong input in SMG and CG from birth), while Hellenic Greek children are arguably as close to monolingual Greek speakers in Cyprus as possible (SMG-only input from birth), though with considerable exposure to the local variety (CG)—again, certainly, once they start formal schooling.

Just as language development in bilingual children should be compared to that of monolinguals, different language combinations in bi- and multilingual children should be taken into consideration as well. Let us call this approach “comparative bilingualism,” although in a very different conception from occasional mentions in the literature that deals largely with societal and educational issues in bilingualism (cf. Bernbaum, 1979; Baker, 1996). In the next section, we will present our research group's findings on the acquisition and subsequent development of object clitic placement by bilectal and multilingual children in Cyprus. Looking at the four purported dynamic metrics of assessment, we may not yet know how much Greek *input* the bilingual children in Cyprus receive, and how SMG-like it is (which also holds for the bilectals). The same goes for the age of *onset* of SMG, if indeed prior to formal schooling, or the exact role of CSG in this respect. However, we do know for *timing* that object clitics appear very early in Greek (for SMG see Marinis, 2000, and for CG Petinou and Terzi, 2002, as well as our own CAT lab research reported below). And lastly, with respect to language *proximity*, CG as a “dialect” of Modern Greek is by definition very close to SMG (as opposed to, say, Russian). A valuable tool for further teasing apart timing and proximity from onset and input is Tsimpli's (2003) Interpretability Hypothesis (cf. Tsimpli and Mastropavlou, 2007), which has recently been assessed for Russian–Greek-speaking adults residing in Cyprus (Karpava, 2014), though we do not yet have comparable data from Russian-speaking bilingual children growing up in Greece (with SMG), which is part of our ongoing research activities: There does not seem to be a correlation between age/onset/input and the production of clitics, for example, which express uninterpretable features—and for which native-like attainment cannot be reached.

## The CAT clitic corpus: Acquisition and development of clitic placement

One of the best studied grammatical differences between the two varieties pertains to clitic placement (see Agouraki, 1997, and a host of research since): Pronominal object clitics appear postverbally in CG, with a number of syntactic environments triggering proclisis, while SMG is a preverbal clitic placement language in which certain syntactic environments trigger enclisis. In both varieties of Modern Greek, 3rd person object clitics are derived from strong pronouns; clitics are marked for number (singular, plural), gender (masculine, feminine, neuter), and case (accusative, genitive). Concerning the particular characteristics of mixed clitic placement, it can be observed that certain syntactic environments enforce preverbal placement—otherwise enclisis is found. Therefore, clitics in CG can appear postverbally in both imperative and non-imperative contexts, whereas in SMG they can only appear as enclitics in imperatives and gerunds.

**Figure 1:**
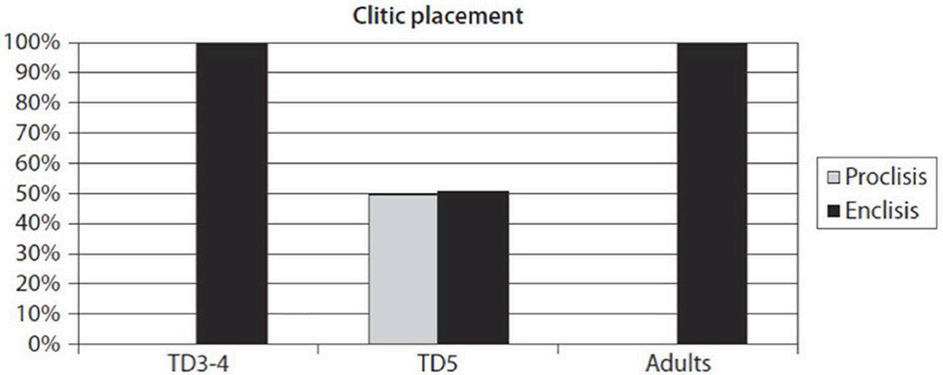
**Clitic placement in clitics-in-islands task (all tested groups)**. From Grohmann (2014a, p. 196).

Now, the acquisition of pronominal clitics is arguably a “(very) early phenomenon,” as Tsimpli (2014) calls it, since clitics represent a core aspect of grammar and are fully acquired at around 2 years of age. Using a sentence completion task that aimed at eliciting a verb with an object clitic in an indicative declarative clause (Varlokosta et al., 2015), we counted children's responses to the 12 target structures in CG, which should consist of verb–clitic sequences (as opposed to clitic–verb in SMG). Methodology and participant details will be provided below. To anticipate the presentation of results, the main pattern is consistent with the one originally reported for our first pilot study (Grohmann, 2011), which was confirmed and extended to many more participants in subsequent work (summarized in Grohmann, 2014a). This pattern is provided in Figure 1.

With very high production rates in all groups (over 92%), the pilot study showed that the 24 three- and four-year-old children behaved like the 8 adult controls: 100% enclisis in the relevant context. In contrast, the group of 10 five-year-olds showed mixed placements, where that group is split further into three consistent sub-groups. The following introduces in some detail the CAT Clitic Corpus of data we have collected to date and briefly presents the main tool(s) used to elicit the responses (from Grohmann, 2014a). This level of detail also underlines our urge for more carefully controlled experimental investigations in the future (picked up in the Discussion and Outlook section). There are numerous references to our published works which each only consider smaller sub-groups; unfortunately, we cannot provide the overall analysis here, since it has not yet been published (Grohmann et al., submitted). For this reason, the presentation of the results below will be rather short and general, but the direction where this research project is heading and the relevance to the present contribution should become clear.

If we only consider the typically developing bilectal Greek Cypriot children that participated in the studies reported in Grohmann (2014a), we currently have 623 datasets of 12 target structures each; for these, we also have 34 adult controls and 20 teenagers, and we can compare them to additional populations, all residing in Cyprus: bilectal children with atypical language development (SLI), bilingual or rather bilectal bilingual children (Russian–Greek), Hellenic Cypriot or binational children (SMG and CG), and Hellenic Greek children and adults (SMG). These groups yield a total of 787 individuals that participated in the clitics tool(s). Most of these were Greek Cypriot children, but there are a number of other participants, as just listed. Likewise, most testing was done on the Clitics-in-Islands tool (COST Action A33, 2006–2010), presented below, but other tasks were used, too (see Grohmann, 2014a, for details and references). Here we focus on reporting data collected on the acquisition and subsequent development of object clitic placement in the two closely related varieties of Greek by bilectal (Grohmann, 2011, 2014a; Grohmann et al., 2012), binational (Leivada et al., 2010), and bilectal bilingual or multilingual children (Karpava and Grohmann, 2014).

As shown in Table 1, this total number of participants breaks down as follows: 727 children from public kindergarten, pre-school, and primary school, 20 teenagers from public middle and high school, and 40 adults from university and the general employment sector, with an eye on gender balance. Of the 727 children, all but 34 had typical language development to the best of our knowledge. 623 were “monolingual” Greek Cypriot children (i.e., *sequential bilectal* in CG and SMG), 40 “monolingual” Hellenic Greek children (native in SMG but exposed to CG due to residence in Cyprus), and 30 binational Hellenic Cypriot children (native in SMG and CG, possibly with a preference for SMG from early on, but otherwise idealized *simultaneous bilectal*). In addition, 18 children were bilectal bilingual (Russian and Greek, i.e., CG and SMG), all with Russian-speaking mothers and Greek Cypriot fathers, but not tested for language delay or impairment, and the remaining 16 bilectal children were diagnosed with SLI by experienced speech–language therapists.

**Table 1:** **Breakdown of all participants (clitic tasks)**.

**Participant groups (ethnicity/language)**	**Number**	**Age range**	**Gender**
Bilectal children (Greek Cypriot/CG and SMG)	6	2;8–2;11	331F, 292M
	23	3;0–3;11	
	154	4;0–4;11	
	193	5;0–5;11	
	185	6;0–6;11	
	36	7;1–7;11	
	26	8;1–8;11	
Monolingual children (Hellenic Greek/SMG)	2	3;2	23F, 17M
	10	4;0–4;10	
	8	5;1–5;11	
	1	6;3	
	12	7;0–7;11	
	7	8;1–8;10	
Binational children (Hellenic Cypriot/SMG and CG)	2	3;7	22F, 8M
	2	4;1–4;2	
	8	5;2–5;10	
	7	6;0–6;11	
	5	7;0–7;10	
	5	8;0–8;7	
	1	9;1	
Bilingual children (Russian–Cypriot/R, CG, SMG)	2	4;8	7F, 11M
	2	5;4–5;6	
	9	6;0–6;8	
	5	7;0–7;8	
Bilectal children with SLI (Greek Cypriot/CG and SMG)	1	4;11	6F, 10M
	8	5;3–5;11	
	1	6;7	
	3	7;1–7;10	
	3	8;1–8;7	
TOTAL (CHILDREN)	727	2;8–9;1	389F, 338M
Bilectal teenagers (Greek Cypriot/CG and SMG)	20	14–18 (mean: 15;6)	11F, 9M
Bilectal adults (Greek Cypriot/CG and SMG)	34	20–65 (mean: 38;6)	17F, 17M
Monolingual adults (Hellenic Greek/SMG)	6	20–30 (mean: 23;6)	2F, 4M

*CG, Cypriot Greek; F, Female; M, Male; SMG, Standard Modern Greek (from Grohmann, 2014a, p. 11)*.

All participants from the studies reported in Grohmann (2011), Grohmann et al. (2012), and Theodorou and Grohmann (2015) were semi-randomly recruited across the urban centers of Nicosia and Limassol. The children from Leivada et al.'s (2010) study all came from the Nicosia municipality, and the bilingual children from Karpava and Grohmann (2014) all grew up in the Larnaca area. Due to the nature of the investigation (see Agathocleous et al., 2014), the children recruited for Agathocleous (2012) and Charalambous (2012) not only came from all over Cyprus (minus Nicosia and Limassol) but were also balanced for urban vs. rural upbringing. The reason for these details lies in the often raised but largely anecdotal claim that there is geographically based dialectal variation in Cyprus and that rural CG differs from urban CG. While this may be the case in many domains of the language (such as, most obviously, the lexicon), it did not seem to make a difference for the clitics task at hand, though in the absence of an empirically grounded knowledge base, we had to go to lengths to determine said absence of effects.

Further prerequisites for child participation included the following (with the exception of the Russian–Greek children from Karpava and Grohmann, 2014): Children had to attend Greek-speaking nurseries or kindergartens, be monolingual (i.e., bilectal) speakers of CG, and not have received speech–language therapy services. They were tested upon written parental consent and with approval from the Cyprus Ministry of Education and Culture (through the Pedagogical Institute). Of the older participants, 20 Greek Cypriot teenagers and 28 Greek Cypriot adults were tested who were all born and raised in Cyprus and resided in Cyprus at the time of testing; none of the teenagers had spent any large amounts of time outside the country. In addition, 6 Hellenic Greek adults residing in Cyprus were tested. None of the older participants was reported to have had speech, language, or communication difficulties.

In sum, what this line of research focuses on is a comparable “linguality” of participants, here children that grow up with one language (Greek) which comes in at least two distinct (i.e., discrete) lects, CG and SMG, leaving aside the issue of CSG. The attribute of linguality goes beyond, or in addition to, whether a child may also grow up bilingually (simultaneously or sequentially) or learn additional languages later on. In the absence of (i) relevant studies concerning quality and quantity of lectal input, age of onset, and other important factors for the early years, as well as (ii) a clear characterization of acrolectal CG as CSG and (iii) its relevance for child language development, we have to leave things here as they stand and idealize somewhat. It is in this sense that we describe the linguality of Greek Cypriots as (discrete) bilectalism.

For the purpose of this research, the COST Action A33 Clitics-in-Islands testing tool (Varlokosta et al., 2015)—originally designed to elicit clitic production even in languages that allow object drop, such as European Portuguese (Costa and Lobo, 2007)—was adapted to CG (from Grohmann, 2011). This tool is a production task for a 3rd person singular accusative object clitic within a syntactic island in each target structure in which the target-elicited clitic was embedded within a *because*-clause (where the expected child response is provided in brackets and the clitic boldfaced):

(1)  To   aγori vre∫i  ti     γata t∫e   i    γata e  vremeni. Jati   i     γata e vremeni?
the  boy   wets the  cat   and the cat   is wet        why  the cat   is wet
I     γata e vremeni  jati        to   aγori… [vre∫i                **tin**].
the  cat  is wet        because the boy      wet.PRES.3SG CL.ACC.3SG.FEM
‘The boy is spraying the cat and the cat is wet. Why is the cat so wet? The cat is wet because the boy…[is spraying it].'

**Figure 2:**
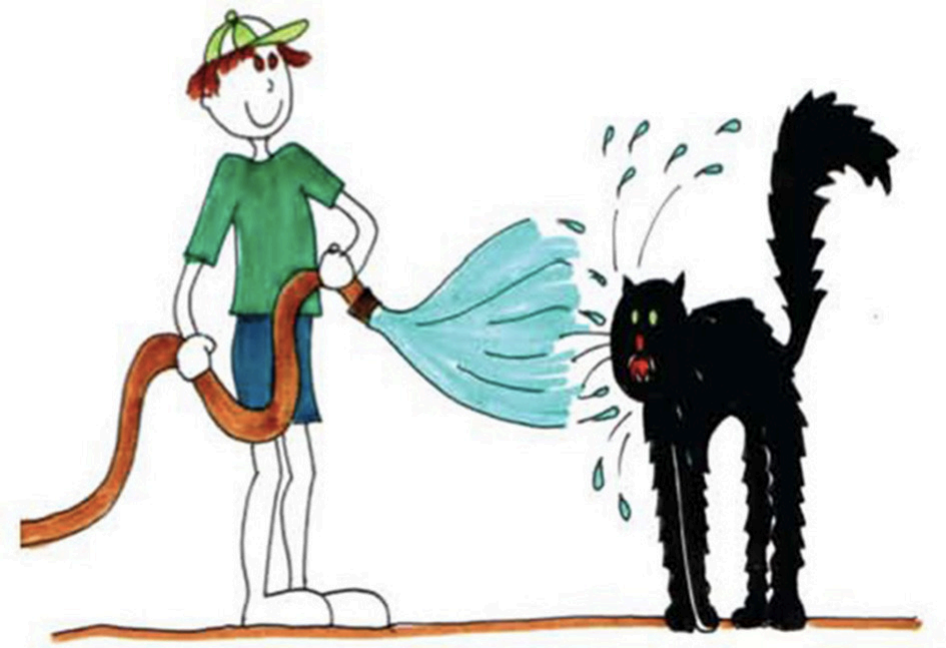
**Sample test item (“long version” and “short version”)**. From Varlokosta et al. (2015).

The task involved a total of 19 items; 12 target structures (i.e., test items) after 2 warm-ups, plus 5 fillers. All target structures were indicative declarative clauses formed around a transitive verb, with half of them in present tense and the other half in past tense. Children were shown a colored sketch picture on a laptop screen, depicting the situation described by the experimenter. The scene depicted in Figure 2 corresponds to the story and sentence completion in (1), for example.

Other test examples can be found in Agathocleous et al. (2014), who also discuss the “short version” in some detail (a pre-version developed within COST Action IS0804, 2009–2013), as well as Karpava and Grohmann (2014), who in addition present the Production Probe for Pronoun Clitics tool (based on Tuller et al., 2011).

Combining the different tasks and participant details, our growing CAT Clitics Corpus—and as yet not fully statistically analyzed beyond what is reported here (though for a first attempt see Grohmann et al., submitted)—at present contains data from a host of participants (Grohmann, 2014a, p. 14). These details are summarized in Table 2, where the boldfaced row indicates the total numbers of participants tested on a comparable tool, namely some version of the above-described elicitation tool for CG with 12 identical target-elicitation structures in either version.

**Table 2:** **Number of participants (per tool)**.

**Clitic elicitation tool**	**Bilectal children**	**Other children**	**Teenagers and adults**
Clitics-in-Islands tool (“long version”: CG)	443 16 (SLI)	18 (BL) 30 (BN) 40 (HG)	10 (GC T) 24 (GC A) 6 (HG A)
Clitics-in-Islands tool (“short version”: CG)	180	—	10 (GC T) 10 (GC A)
TOTAL	623 16 (SLI)	18 (BL) 30 (BN) 40 (HG)	20 (GC T) 34 (GC A) 6 (HG A)
Clitics-in-Islands tool (“modified long”: SMG)	40	30 (BN) 40 (HG)	6 (GC A) 6 (HG A)
Production Probe for Pronoun Clitics tool (CG)	—	18 (BL)	—

*A, adult; BL, bilingual; BN, binational; CG, Cypriot Greek; GC, Greek Cypriot; HG, Hellenic Greek; SLI, specific language impairment; SMG, Standard Modern Greek; T, teenager. From Grohmann (2014a, p. 15)*.

All tests with Greek Cypriot bilectal children were carried out by native speakers of CG; those tests that were administered in SMG were done by a native SMG speaker. Testing was conducted in a quiet room individually (child and researcher). Most children were tested in their schools or in speech–language therapy clinics, but a few were tested at their homes. It is well known that Greek Cypriots tend to code-switch to SMG or some hyper-corrected form of “high CG” when talking to strangers or in formal contexts, as mentioned by Arvaniti (2010), Rowe and Grohmann (2013), and references cited there. For this reason, in an attempt to avoid a formal setting as much as possible (and thus obtain some kind of familiarity between experimenter and child), a brief conversation about a familiar topic took place before the testing started, such as the child's favorite cartoons.

All participants received the task in one session, some in combination with other tasks (such as those tested in Theodorou and Grohmann, 2015; see Theodorou, 2013). The particular task lasted no longer than 10 min, the “short version” even less. The pictures were displayed on a laptop screen which both experimenter and participant could see. The child participants heard the description of each picture that the experimenter provided and then had to complete the *because*-clause in which the use of a clitic was expected; some participants started with *because* on their own, others filled in right after the experimenter's prompt of *because*, and yet others completed the sentence after the experimenter continued with the subject [the bracketed part in (1) above].

No verbal reinforcement was provided other than encouragement with head nods and fillers. Self-correction was not registered; only the first response was recorded and used for data collection and analysis purposes. Regardless of the child's full response, all that was counted were verb–clitic sequences (for clitic production) and the position of the clitic with respect to the verb (for clitic placement). Except for the studies reported in Agathocleous et al. (2014), the experiments were not audio- or video-taped, but answers were recorded by the researcher or the researcher's assistant on a score sheet during the session; many testing sessions involved two student researchers with one carrying out the task and the other recording the responses (in alternating order). In those studies in which different clitic tasks were administered (Karpava and Grohmann, 2014), or where the same tool was tested in CG and SMG (Leivada et al., 2010), participants were tested with at least 1 week interval in between.

## (Discrete) bilectalism and the socio-syntax of language development

All these different studies with different populations and different age groups but the same tool show the following. First, the production rate of clitics in this task is very high from an early age on, safely around the 90% mark from the tested age of 2;8 onwards (lowest production at around 75%), over 95% at age 4;6 (lowest production at around 88%), and close to ceiling for 5-year-olds and beyond. The sub-group of 117 children from Grohmann et al. (2012) performed as shown in Table 3.

**Table 3:** **Clitic production (adapted from Grohmann et al., 2012)**.

**Age range (Number)**	**Overall clitic production (%)**	**Target postverbal clitic placement (%)**
2;8–3;11 (*N* = 26)	89.4	89.2
4;0–4;11 (*N* = 21)	88.5	88.0
5;0–5;11 (*N* = 50)	94.3	68.0
6;0–6;11 (*N* = 20)	87.3	47.0
adult controls (*N* = 8)	100	100

*From Grohmann (2014a, p. 17)*.

This said, Leivada et al. (2010) found considerably higher productions for the younger Hellenic Greek and Hellenic Cypriot children tested compared to their Greek Cypriot peers. However, just considering the 623 bilectal children, we can confirm that the task was understood and elicited responses appropriate; in the widely tested age group of 5-year-olds, the production numbers are among the highest of all languages tested (Varlokosta et al., 2015). High production means reliable data points for all 12 target structures; statistical analysis confirms that there were neither item effects nor test effects, that is, the productions for the “long” and “short” version of the clitics tool are fully comparable (Grohmann, 2014a).

Second, and most importantly, the analysis of the 431 datasets of the bilectal children presented by Grohmann et al. (submitted) are consistent with the findings of the much smaller pilot study (Grohmann, 2011). In other words, Figure 1 can be used as a general indicator: Up to around age 4, children reliably produce enclisis in this task at just shy of 90%, as expected (and confirmed by adult speakers), while we find considerable variation in clitic placement in the 5- to 7-year-olds.

**Figure 3:**
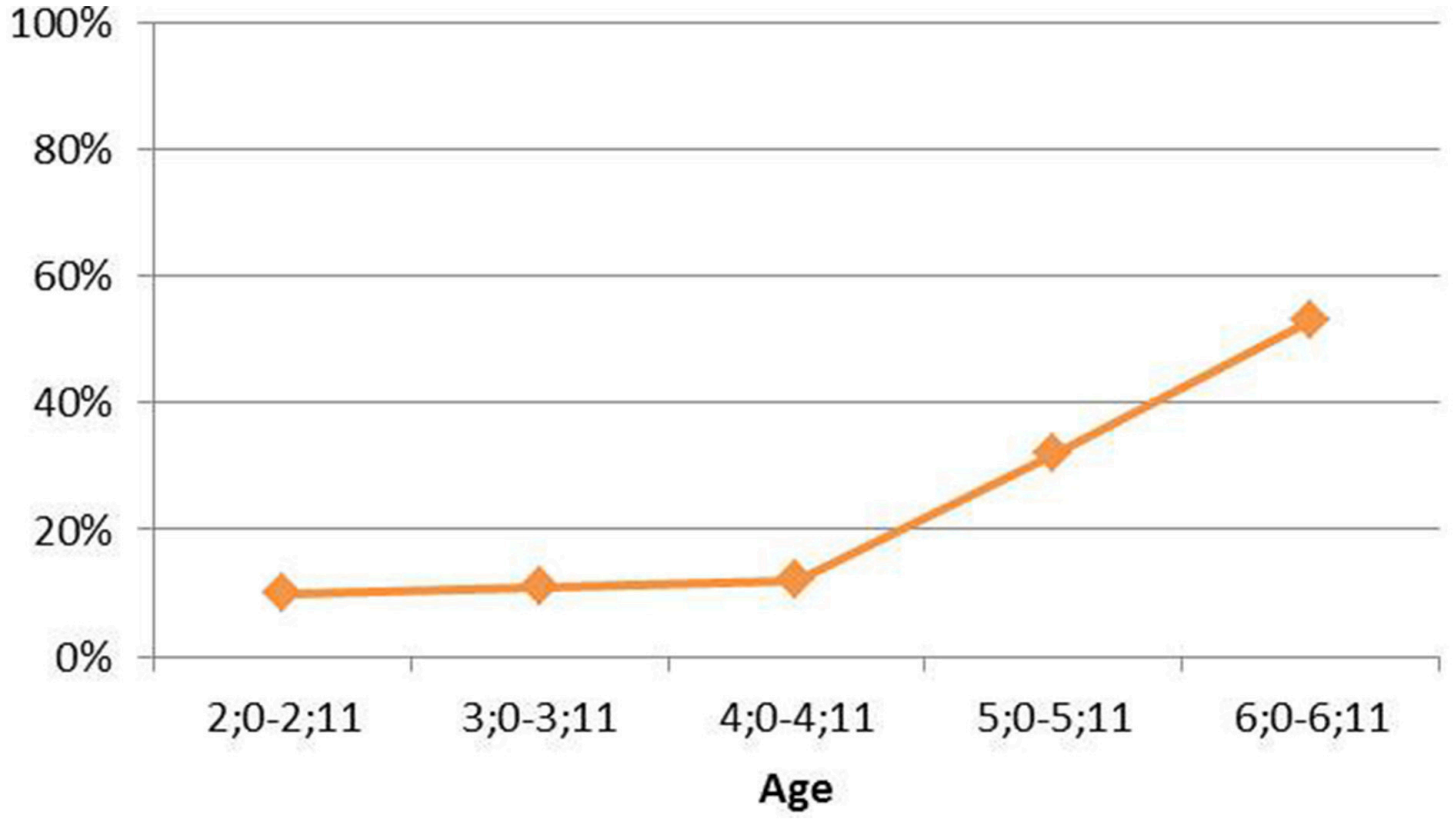
**Non-target preverbal clitic placement (by chronological age)**. The x-axis indicates participants according to their chronological age, while the y-axis plots non-target preverbal clitic placement in the participants' responses (percentage). From Grohmann and Leivada (2011).

To illustrate with the subset of 117 children again, when their non-target preverbal clitic placement productions were plotted according to chronological age, the resulting curve looks as in Figure 3.

**Figure 4:**
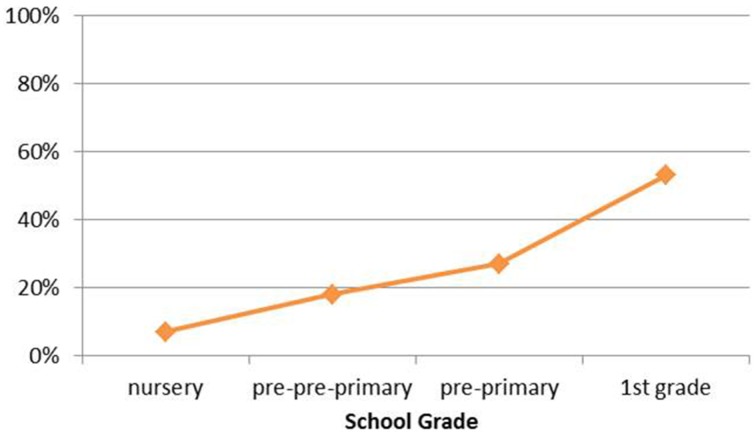
**Non-target preverbal clitic placement (by schooling level)**. The x-axis indicates participants according to their schooling level, while the y-axis plots non-target preverbal clitic placement in the participants' responses (percentage). From Grohmann and Leivada (2011).

However, what we can observe are apparent inconsistencies in terms of clitic placement, in particular by comparing younger with older children according to their schooling level. While for nursery children (mean age 3;3), target postverbal clitic placement lies at 93%, it decreases systematically for each additional year of formal schooling: kindergarten (4;3) at 82%, pre-school (5;5) at 73%, and first-grade (6;7) at 47%—from grade 2 onwards, the rates quickly shoot up toward 100% again (Grohmann, 2014a). This analysis is extended in Grohmann et al. (submitted). But using the same sub-group of 117 children again, compare Figure 3 above with Figure 4.

The most striking result is that, while at the youngest ages, prior to formal schooling, the CG-target enclisis is produced predominantly, if not exclusively, once Greek Cypriot children start getting instructed in the standard language (SMG or some such equivalent like CSG), their non-target productions of proclisis rise dramatically—all the way to second grade (not shown here; full analysis provided in Grohmann et al., submitted).

One obvious way to approach the situation is to appeal to “competing grammars.” Kroch (1994: 180) proposes competition of grammatical systems for diachronic change in that “syntactic change proceeds via competition between grammatically incompatible options which substitute for one another in usage” (for specific accounts and extensions to language acquisition models, e.g., Kroch and Taylor, 2000; Yang, 2000; Legate and Yang, 2007). Following Lightfoot's (1999) description of competing grammars reflecting “internalized diglossia,” this might indeed be a good approach to take up for CG. In fact, Tsiplakou (2009, 2014) had already addressed a possible implementation of the competing-grammars hypothesis for CG; for further discussion, as well as the extension to the older notions of “competing motivations” (Du Bois, 1985) and “metalinguistic awareness” (Cazden, 1976, see Leivada and Grohmann, 2016).

Such an approach would pit the native CG grammar (in this case: enclisis) against the emerging SMG grammar (here: proclisis), which happens to grow stronger through increased input. Since formal schooling is carried out, by law, in the medium of SMG, it is around the entrance into the public schooling system that the SMG grammar becomes stronger, perhaps even dominant at times. This does not imply, however, that public schools in Cyprus would constitute a monolingual, monodialectal environment for pupils. Classroom studies have shown that “CG is very often used as a medium of interaction and even instruction during classroom,” as a reviewer reminded us, across all grades (e.g., Yiakoumetti, 2007; Sophocleous and Wilks, 2010; Sophocleous, 2011).

We would like to take these findings one step further and suggest that they are best captured by the *Socio-Syntax of Development Hypothesis* (Grohmann, 2011), namely that an explicit “schooling factor” is involved in the development of the children's grammar. Note that this grammatical development takes place past the critical period and does so possibly in combination with “competing motivations” (Grohmann and Leivada, 2011; Leivada and Grohmann, 2016). These arguably stem from the (at least) two grammars in the bilectal child's linguistic development that compete with each other. In other words, the Socio-Syntax of Development Hypothesis can be seen as the specific trigger for competing grammars in the development of CG clitic placement by young children.

A way to appreciate the more general Socio-Syntax of Development Hypothesis would be to approach the acquisition of syntactic variants, which CG enclisis and SMG proclisis in the same environment arguably are, by assuming competing motivations that arise between the home and the school variety. In the present case, CG enclisis competes with SMG proclisis in the same syntactic context between two varieties in a dialectal continuum which thus have close proximity. Given that all schooling is done through the medium of SMG, the relevant competing motivations in Cyprus may derive from the absence of bilectal education that could increase children's awareness of the low social prestige of their native CG (see also Rowe and Grohmann, 2013, for further discussion and references).

Note that the rate of 100% proclisis in the Hellenic Greek children is by no means an accidence. A study carrying out the identical tool in Greece (Varlokosta et al., 2014) found that children aged between 3;6 and 5;11 as well as children with SLI exclusively produced proclitic placement of the direct object clitics—as was expected, since SMG does not allow for enclisis in the environment tested (also reported in Varlokosta et al., 2015). A similar point can be made for the binational Hellenic Cypriot children, who performed more like the Hellenic Greek children (in Greece and Cyprus) than their Greek Cypriot bilectal peers. Here we might find a possible difference in development for simultaneous vs. sequential bilectals: If on the right track, Hellenic Cypriot children, having simultaneously acquired CG and SMG, do not enter into competition due to confusion or increased SMG input; both varieties are perfectly natural sources of linguistic input from birth. In addition, as fully balanced users of both, they do not enter competing motivations either but are already metalinguistically aware of the two systems and their appropriate use. (See also the next section for added evidence coming from cognitive abilities, though Hellenic Cypriot children need yet to be assessed, which is part of an ongoing dissertation under the first author's supervision).

Lastly, we also collected data from a group of clear-cut bi- or multilingual children in Cyprus: Russian–Greek speakers, particularly those with a Russian-speaking mother and Greek Cypriot father, whose languages are thus Russian, CG, and SMG (Karpava and Grohmann, 2014); in fact, these children are perhaps best labeled “bilectal bilingual.” Comparing our data from 18 bilectal bilingual children on the same tool with 40 bilectal children (Leivada et al., 2010), we note the following stark contrast in target postverbal clitic placement (with almost identical production levels): kindergarteners at only 22% enclisis (SD 2.08, compared to 82% for bilectals), pre-schoolers at 8% (SD.71, compared to 73%), and first-graders at 11% (SD 3.26, compared to 47%).

Clitic placement thus shows that the bilingual children increased their usage of proclisis and decreased enclisis from kindergarten to primary school. In contrast to the bilectal children, they exhibited much more proclisis than target enclisis early on. This may be due to the additional presence of SMG in the family environment rather than CG-only: Due to L2 learning through formal instruction, most of the Russian mothers' input when addressing their child in Greek (which is quite frequent) would be more SMG-like. In addition, they tend to have a negative attitude toward CG. Since the bilingual children also have higher metalinguistic awareness, they are influenced by their mothers as well as their peers: The former often exhibit a negative attitude toward the CG variety, while the latter arguably show a strong preference toward it. At school, they are forced to use SMG, which is in line with their mothers' linguistic behavior, but contrasts with their peers' and their fathers'. In this sense, they are constantly urged to not only make a choice of language (Russian vs. Greek), but also of variety (CG vs. SMG), and this choice seems to be influenced by different factors.

Let us phrase this in the context of Tsimpli (2014). While clitic acquisition in terms of production is not a problem for simultaneous bilingual children, the appropriate use is somewhat more tricky. First, it is known that non-core aspects of language license the appropriate use and interpretation of clitics, such as pragmatics and discourse/context sensitivity. These are particularly relevant for bilingual populations who acquire a clitic language alongside a non-clitic language (such as Russian), for which the appropriate referent choice is often at stake (full DP vs. strong vs. clitic pronoun), as Parodi and Tsimpli (2005), among others, have shown. In addition, we are dealing with a different situation which lies clearly outside core grammar: the sociolinguistically appropriate placement of clitics. We observe that both bilectal and bilingual children struggle with the context-appropriate form, which arguably involves a certain amount of maturation and metalinguistic awareness.

## A gradience of the cognitive advantage of bilingualism?

We will now turn to a first study on the purported bilingual status of Greek Cypriot bilectal children and its relevance for a more gradient, comparative bilingualism. The results from a range of executive control (EC) tasks administered to monolingual SMG-speaking children (in Greece) as well as CG–SMG bilectal and Greek–English bi-/multilingual children (in Cyprus) suggest that bilectal children behave more like their multilingual rather than their monolingual peers (Antoniou et al., 2014)—that is, on a scale in between.

It has frequently been suggested that bilingualism bears an impact on children's linguistic and cognitive abilities (see recent overviews by and the literature cited in Kroll and Bialystok, 2013; Barac et al., 2014). For example, as already mentioned above in the context of Tsimpli (2014), bilingual children arguably have smaller vocabularies in each of their spoken languages as a result of input deficit (e.g., Paradis and Genesee, 1996; Oller and Eilers, 2002; Unsworth, 2013). On the other hand, bilingual children seem to exhibit earlier development of pragmatic abilities: They are more advanced in computing scalar implicatures (Siegal et al., 2007) and better in detecting violations of Gricean maxims (Siegal et al., 2009, 2010), for example; bilingual children presumably compensate for their lower lexical knowledge by paying more attention to contextual information. And then there is the long-standing claim that bilingualism enhances children's development of EC, the set of cognitive processes that underlie flexible and goal-directed behavior, commonly referred to as the “bilingual advantage” or “cognitive advantage of bilingualism” (for overviews, e.g., Bialystok, 2009; Baum and Titone, 2014; Costa and Sebastián-Gallés, 2014; see also the meta-analysis provided by Adesope et al., 2010). Taking a particular influential one of the many approaches to EC, there is a tripartite distinction into working memory, task-switching, and inhibition (Miyake et al., 2000), each with their own rationale, though more recently some doubt has been cast on inhibition as a separate executive component (Miyake and Friedman, 2012).

Starting with the latter, a bilingual advantage in inhibition may relate to the ability to suppress dominant, automatic responses or irrelevant information (e.g., de Abreu et al., 2012; Poarch and van Hell, 2012). There is also some evidence for advanced task-switching, that is, the ability to flexibly switch attention between rules (e.g., Bialystok and Viswanathan, 2009; Foy and Mann, 2014). The effect of bilingualism on working memory, the ability to simultaneously maintain and manipulate information in mind, is more controversial, however (e.g., de Abreu, 2011; Morales et al., 2013; Blom et al., 2014; Calvo and Bialystok, 2014).

This composite approach to EC is arguably superior to an earlier suggestion that the bilingual advantage can be traced exclusively to more advanced inhibition alone (e.g., Bialystok, 2001). Here the idea was that, because both linguistic systems are activated when a bilingual speaks in one language, fluent use requires the inhibition of the other language. This constant experience in managing two active conflicting linguistic systems via inhibition enhances bilinguals' inhibitory control mechanisms. This early view, however, has been challenged on several grounds (e.g., Bialystok et al., 2012). One line of argument would be that advantageous effects of bilingualism have been observed for the very first years of life, even for 7-month-old infants (Kovács and Mehler, 2009). Since language production has not yet started in bilingual infants, there would be no need to suppress a non-target language. We are not sure that this argument goes through, though: After all, even bilingual infants are fully aware of the different languages they are acquiring, and while they may not need to inhibit one to produce the other, they presumably process the two (or more) languages and should therefore regularly inhibit one to process the other. However, there are a number of further arguments to take a more differentiated view on EC as the measuring stick for the bilingual advantage, as put forth in many of the references cited above; see also Antoniou (2014) and Antoniou et al. (2016) for further discussion.

All in all, an advantage in EC may be the result of constantly having to manage two different linguistic systems. One aspect of continued research on the topic would thus be to disentangle the different EC sub-components and determine which aspect(s) of executive control really relates to a bilingual advantage. Regarding performance on executive control in monolingual, bilectal, and bi- or multilingual children, the relevant research question is then (Antoniou et al., 2014): What is the effect of bilectalism on children's vocabulary, pragmatic, and EC skills?

A total of 136 children with a mean age of just above 7.5 years of age participated in the study (Antoniou et al., 2014): 64 Greek Cypriots, bilectal in CG and SMG, aged 4;5–12;2 (mean age: 7;7, SD: 1;6 years; 32 boys, 32 girls); 47 residents of Cyprus, multilingual in CG, SMG, and English (plus in some cases an additional language), aged 5;0–11;5 (mean 7;8, SD 1;8; 24 boys, 23 girls); and 25 Hellenic Greeks, monolingual speakers of SMG, aged 6;2–9;0 (mean 7;4, SD 0;9; 15 boys, 10 girls). Socio-economic status measures included the Family Affluence Scale (Currie et al., 1997), while level of maternal and paternal education, among other details, were obtained through questionnaires (Paradis et al., 2010; Paradis, 2011). Since the multilingual children all attended a private English-medium school, their socio-economic was higher than the mean of all other participants.

A range of language proficiency measures were administered for expressive and receptive vocabulary, including the Greek versions of the Word Finding Vocabulary Test for expressive vocabulary and the revised Peabody Picture Vocabulary Test (SMG) as well as the Greek Comprehension Test (for either variety). For pragmatic performance, a total of six tools were used, tapping into relevance, manner implicatures, metaphors, and scalar implicatures; the bilectal and multilingual children received the test in CG, the bilectals took the test in both CG and SMG, and the monolinguals were tested in SMG only. As for non-linguistic performance, the WASI Matrix Reasoning Test was used to assess participants' non-verbal intelligence. The EC tasks administered included a wide range of batteries. For verbal working memory, the Backward Digit Span Task was employed, and for visuo-spatial working memory, an online version of the Corsi Blocks Task. Inhibition was assessed through Stop-Signal and the Simon Task, and switching through the Color–Shape Task. (For more details and references, see Antoniou, 2014; Antoniou et al., 2014.) In the end, we opted for a composite measure of EC which was computed in a principled component analysis for the factors Working Memory and Inhibition over the individual results (Antoniou et al., 2016).

The analysis results from a two-stage comparisons between the three groups. First, the performance of all child participant groups was compared to each other (monolinguals vs. bilectals vs. multilinguals); the three groups were matched in age by excluding all children who were below 6 and above 9 years of age. Then the performance of a subset of 17 bilectal children was compared to that of the monolingual group. All these children were also administered a receptive vocabulary test in order to test whether exercising a more rigid statistical control over children's language skills would reveal or increase potential bilectal advantages in EC. As Antoniou et al. (2016) show, the two composite measures (Working Memory and Inhibition) significantly and positively correlate with language ability; also, the bilectal children were possibly disadvantaged in language proficiency relative to monolinguals.

The results from this study can be presented across four types of group comparisons. The first concerns background measures. The relevant subsets of the three participant groups of bilectal (*n* = 44), multilingual (*n* = 26), and monolingual children (*n* = 25) aged 6;0–8;11 were intended to be matched for age and gender; they did not statistically differ on age [*F*_(2, 92)_ = 0.696, *p* > 0.05] or gender [*F*_(2, 92)_ = 0.587, *p* > 0.05]. However, they did differ on socio-economic status [*F*_(2, 89)_ = 9.622, *p* < 0.05], with the private-schooled multilingual children as a group coming from a higher socio-economic family background than the monolingual ones, and the bilectals from the lowest. The three groups also differed on non-verbal IQ [*F*_(2, 92)_ = 3.377, *p* < 0.05], with the multilingual children higher than the two other groups, which did not differ significantly.

Next we compared the three participant groups' performance on the vocabulary measures. The multilingual children had a significantly lower vocabulary score than the bilectals, who in turn had a significantly lower vocabulary than the monolinguals [*F*_(2, 92)_ = 44.183, *p* < 0.05], confirmed by *post-hoc* pairwise comparisons with Bonferroni correction for multiple comparisons (all *p*s < 0.05). From what is known about vocabulary growth in bilingual contexts (see references above), it was expected that the monolingual children would outperform the multilinguals; the fact that the bilectals fall in between fits nicely with our hypothesis that, on a gradient scale, bilectalism lies somewhere in between mono- and multilingualism.

The third group comparison concerns performance in the pragmatic tasks (Antoniou et al., 2014; this is not part of the extended analysis reported in Antoniou et al., 2016). Analyses of covariance (ANCOVAs), with vocabulary and SES & IQ as covariates, showed no significant differences between the three groups across all pragmatics tasks [*F*_(2, 87)_ = 4.081, *p* < 0.05]. No differences in the pragmatic tasks suggest that even those children who exhibit some sort of lower language (multilinguals, perhaps bilectals), they still show comparable pragmatic performance at the same age. With an eye on the Greek Cypriot bilectal children, this again suggests that they pattern somewhere in between; given the lower vocabulary scores compared to their monolingual peers from Greece, they do perform the same in the six pragmatic tasks.

Lastly, and for the purposes of our research question perhaps most importantly, the child participants' performance on the EC tasks was analyzed and submitted to principal component analysis (Antoniou et al., 2014). All three global EC scores (working memory, inhibition, and switching) positively correlated with IQ. ANCOVAs on the three composite scores for EC, with Group as a between-subjects factor and IQ, linguistic knowledge (Greek), age, and SES as covariates, revealed a significant effect of group only for the overall EC score: a significant multilingual advantage over monolinguals, with a trend for a bilectal advantage.

We illustrate this finding here with switch cost from the original analysis (Antoniou et al., 2014): Bilectals performed better than monolinguals in the congruent switch trials, with no other significant comparisons [*F*_(2, 87)_ = 4.081, *p* < 0.05]; in the incongruent switch trials, bilectals also performed better than monolinguals [*F*_(2, 87)_ = 5.805, *p* < 0.05], with multilinguals almost better than monolinguals (*p* = 0.108). These results can be summarized as showing that the bilectal children performed better than the monolinguals in overall EC ability and slightly worse than multilinguals. With respect to the lack of a clear effect for switching, as opposed to vocabulary, for example, we would like to suggest that there is an interference from language proximity: The more similar the two varieties, the more difficult it is to switch—or rather, the less there is a need to switch. For example, in a given group of individuals of whom all but one speak Greek and English, with one knowing no Greek, a Greek-language discussion would be translated or summarized in English for that individual [switching by the bilingual speaker(s)]. In contrast, in a group of Greek speakers of whom only one does not speak Cypriot Greek, a CG-at large discussion would arguably not be translated or summarized in SMG for that individual [no switching by the bilectal speaker(s)]. As noted in a different context by Runnqvist et al. (2012), this may in fact tie in with the reverse of a bilingual advantage, what they call the “bilingual disadvantage.” Beyond the cases they examine (e.g., Ivanova and Costa, 2008; Costa et al., 2009), it has also been suggested that the cognitive advantage only surfaces in bilingual individuals who actually switch between their languages frequently (Prior and Gollan, 2011).

In the extended statistical analysis of Antoniou et al. (2016), it could furthermore be shown through a mixed ANCOVA that, while the Working Memory and Inhibition composite scores significantly correlated with IQ, general language ability in Greek, and age, multilinguals had a significantly higher EC performance than monolinguals (*p* < 0.05), without any significant differences between the other groups (all *p*s > 0.05, Bonferroni correction applied). Also, since the Group × EC interaction was not significant [*F*_(2, 84)_ = 0.744, *p* > 0.05], the multilingual advantage in EC was not specific to Working Memory or Inhibition. Moreover, the second stage of the statistical analysis explores the possibility that a bilectal advantage over monolinguals can indeed be found if children's language proficiency in Greek is more rigidly controlled (see Antoniou et al., 2016).

In terms of a larger discussion, we hasten to add that there is recent work that casts some doubt on the purported relation between bilingualism and EC abilities (e.g., Paap and Greenberg, 2013; Paap and Sawi, 2014). Just like the above-mentioned modifications to the “right” kind of model of EC, there are a number of factors that make more careful investigations even more important. In the study reported here (Antoniou et al., 2014, 2016), for example, we compared group performances. However, the groups were composed of rather few children of a considerable age range, and, for obvious reasons for the populations chosen, there were significant differences in socio-economic status and non-verbal intelligence. Likewise, it is not yet clear in how much, if at all, the cognitive advantage observed in bilingualism pertains or increases in multilingualism. These are some of the considerations that our future work aims to improve in order to assess the purported bilingual advantage in EC abilities in bilectal speakers as well as finer grained and better selected multilingual groups for comparison.

An associated extension of the “bilingual advantage” in cognitive development for closely related varieties concerns children's development of literacy skills. This issue has recently been addressed for the two Norwegian literary varieties, Nynorsk and Bokmål, by Vangsnes et al. (2015). Although not directly linked to EC abilities, there is a growing body of work on literary development in Cyprus (Tsiplakou, 2006; Hadjioannou et al., 2011), but more recent research from Greece for SMG connects EC abilities explicitly with literary skills for mono- and bilingual children (Andreou, 2015; Andreou and Tsimpli, submitted). This connection is currently being investigated for bilectal, bilingual, and monolingual children at CAT as part of an ongoing dissertation under the first author's supervision.

## Lessons from developmental language impairment

In this third line of research related to the role of bilectalism within a comparative view to bi- or even multilingualism, we shift to studies that focus on the manifestations of lexical retrieval or spoken naming breakdown in atypical and impaired language development. The data reported come from our growing CAT Naming Corpus which includes monolingual, bilectal, bilingual, and multilingual child speakers of Greek. Here we aim to highlight the relevance of this research for a more gradient, comparative perspective of bilingualism in the context of developmental language impairments.

Lexical retrieval deficits, or childhood anomia, are a frequent part of the symptom complex that characterizes children with language impairments and are usually defined as “delayed or inaccurate responses with a high incidence of repetitions, reformulations, word substitutions, insertions, time fillers, and empty words” (German and Newman, 2004, p. 624). Speech and language therapists working with language-impaired children with anomia report co-existing impairments in other linguistic (expressive language, phonology, literacy) and non-linguistic domains (e.g., working memory); recent up-to-date reviews can be found in Friedmann et al. (2013) and Kambanaros et al. (2015). Depending on the severity, anomia may have severe repercussions for children in school settings, the significance being that classroom communication and academic skills, including reading and writing, are usually adversely affected (see Messer and Dockrell, 2011). Moreover, when anomia impedes communication with peers and others, children's psycho-social well-being is shown to be compromised (Tomblin, 2008). The emphasis lies on difficulties with lexical retrieval that manifests as an inability to name things like concrete entities (named by nouns) and actions (named by verbs).

We report on a study where the performance of multilingual children with SLI residing in Cyprus was compared with the performance of a language-matched group of multilingual children without SLI and with bilectal children, with and without SLI, on the same task. Multilingual children are in this context defined as children who simultaneously acquire two first native languages (e.g., CG and English) and SMG as a third language upon entering the school system, usually by the age of four (hence possibly falling under early second language acquisition; see Meisel, 2007); alternatively, one might refer to them as “bilectal bilinguals.” The task used was a picture-based naming test of concrete noun and verbs, the Cypriot Object and Action Test (COAT; Kambanaros et al., 2013b). For a subgroup of the multilingual children with SLI, performance on noun and verb naming was investigated in two spoken languages (namely, Greek–English), using the English version of the OAT (Kambanaros, 2003, 2013).

A total of 59 children participated in the noun–verb naming study, divided into four groups:

- *bilSLI* (*n* = 14): 14 bilectal children with SLI (4 girls and 10 boys), aged 5;5–9;9 (average age 6;9, standard deviation 1;8)
- *multiSLI* (*n* = 5): 5 multilingual children with SLI (2 girls and 3 boys), aged 6;6–9;2 (mean age 7;11, standard deviation 1;1)
- *bilTLD-LM* (*n* = 30): 30 typically developing bilectal first-graders (15 girls and 15 boys), aged 6;0–6;11 (mean age 6;3, standard deviation 0;3), serving as the language-matched bilectal group for multilingual children with SLI
- *multiTLD-LM* (*n* = 10): 10 typically developing multilingual children (7 girls and 3 boys), aged 4;6–6;11 (mean age 5;1, standard deviation 0;9), serving as the language-matched control group for the multilingual children with SLI.

The children with typical language development (both, bilTLD-LM and multiTLD-LM) were recruited randomly from three public primary schools in urban Cyprus after approval from the Ministry of Education and Culture, and upon written parental consent. No typically language-developing child was or had ever been receiving speech–language therapy services. The children with SLI (both, bilSLI and multiSLI) were recruited from speech and language therapists in public primary education and/or private practice. All language-impaired children were in mainstream education and in the school grade corresponding to their chronological age. Also, they had received or were receiving speech–language therapy and/or special education services separate from their classmates and the regular classroom (“pull-in/out service model”).

Subject selection criteria included: no history of neurological, emotional, or behavioral problems, no gross motor difficulties, hearing and vision adequate for test purposes, normal articulation, normal performance on screening measures of non-verbal intelligence (a score no less than 80 on the Raven's Colored Progressive Matrices or as reported by the school psychologist). All children came from families with medium to high socio-economic status. The bilectal children (both, bilTLD-LM and bilSLI) came from a Greek Cypriot background, with exposure to CG as the exclusive home language and SMG as the language of schooling. For the multilingual children (both, multiTLD-LM and multiSLI), a thus-defined bilectal background was required plus early exposure to a third non-Greek language in the home (such as English); in addition, all language acquisition involved bona fide multilingualism (e.g., a child exposed to CG and English from birth and later to SMG at school).

Of the five simultaneous multilingual children with SLI tested, three came from a CG–English language background, one was a CG–Romanian multilingual, and the other CG–Arabic. According to parental reports, all five multiSLI children were Greek-dominant. The group of multiTLD-LM, the typically developing multilingual preschoolers serving as the language-matched control group to the multilingual SLI group, were simultaneous bilinguals of CG (L1a) and a second language (L1b)—here: English, Romanian, Russian, and Arabic—and had acquired SMG as their L2 upon school entry (e.g., kindergarten at 4 years of age). In all cases, the father was of Greek Cypriot background and the mother a native speaker of the non-Greek language just specified. For all participating multilingual children, the Developmental and Language Background questionnaire developed in COST Action IS0804 (2009–2013), which both authors participated in, was given to the mothers to complete (see Tuller, 2015). Further information can be obtained from the authors.

Participating bilectal SLI or bilectal language-control children were not receiving additional instruction in other languages taught in schools (for the former because of their language impairment and for the latter because of their age/grade in school). This allowed us to control for the languages the children were exposed to and propose a homogeneous group, as far as possible, in relation to language exposure and use. Prior to the study, the children with SLI were assessed on a large test battery by certified speech and language therapists, including the second author. To qualify, children had to score lower than the normal range on the standardized tests in Greek in two (or more) linguistic domains. The typically language developing children serving as language-matched controls were matched with the multilingual SLI group based on scores from the standardized Greek version (Vogindroukas et al., 2009) of the Renfrew Word Finding Vocabulary Test (Renfrew, 1997).

Demographic information of the participants and results of the SLI and TLD groups on our language battery are presented in Table 4.

**Table 4:** **Performance on background measures (by group)**.

		**TLD scores (SD)**		**MultiSLI scores (SD)**	**bilSLI scores (SD)**
Raven's matrices		94.58 (9.64)		90 (10)	85.4 (8.89)
DVIQ—Production of Morphosynta	TLD scores are the mean results of administrating the test to a subset of 16 bilTLD children aged 4;6–9;11	19.9 (2.11)		16.8 (4.66)^*^	12.3 (2.09)^*^
DVIQ—Comprehension of Morphosyntax		26.4 (2.46)		22.6 (4.28)^*^	22.4 (1.84)^*^
DVIQ—Sentence Repetition		46.8 (1.80)		42.8 (2.77)^*^	40.8 (2.70)^*^
DVIQ—Vocabulary		22.3 (1.58)		19.04 (2.41)^*^	15.7 (2.20)^*^
DVIQ—Metalinguistic abilities		20.1 (2.45)		19.8 (1.79)	17.5 (1.29)
Word Finding Vocabulary Test (WFVT)	Norms (in Greek) for children aged 5;1–8;0	26.5–33.2	30.2 (7.9)	24.4 (4.77)	27.5 (4.96)
	10 multiTLD (mean age 5;1)		23.6 (5.87)		

* *Impaired; bilSLI, bilectal SLI; multiSLI, multilingual SLI; SD, standard deviation; TLD, typical language development*.

**Figure 5:**
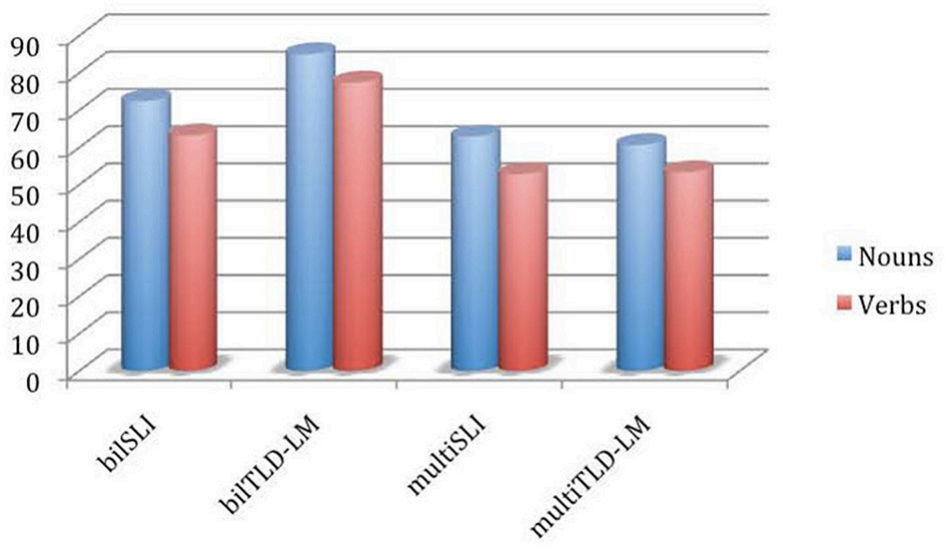
**Percentage of naming accuracies for nouns and verbs (all participants)**. bilSLI, bilectal specific language impairment; bilTLD-LM, bilectal typical language development–language match; multiSLI, multilingual specific language impairment; multiTLD-LM, multilingual typical language development–language match. Modified from Kambanaros et al. (2013a, p. 71).

At a glance, the results from the two clinical and the two control groups can be depicted as in Figure 5.

The four groups were simultaneously compared on the two dependent variables (percentage correct on nouns and percentage correct on verbs), using the non-parametric Kruskal-Wallis test, which revealed significant mean differences on noun and verb accuracies [$\chi_{\left(3\right)}^{2}=18.132$, *p* < 0.001 and $\chi_{\left(3\right)}^{2}=27.422$, *p* < 0.001, correspondingly]. Pairwise comparisons of the multiSLI group with the other three groups were conducted with Mann-Whitney *U*-tests, adopting a Bonferroni adjusted level of significance (0.05/3 = 0.017). When naming accuracies for verbs and nouns of the multiSLI group were compared with the performance of the bilSLI children, the difference was not statistically significant for either word class (z = −0.604, *p* = 0.546 for nouns and *z* = −0.698, *p* = 0.485 for verbs). Similarly, when performance of the multiSLI group was compared to that of their multiTLD-LM peers, there was not a statistically significant difference in naming nouns (*z* = −0.123, *p* = 0.902) or verbs (*z* = 0, *p* = 1). Also, the multiSLI group scored considerably lower than the bilTLD-LM, but the difference failed to reach the adjusted level of significance (*z* = −2.185, *p* = 0.029 for nouns; *z* = −2.081, 0 = 0.037 for verbs).

For the multilingual groups in particular, a Wilcoxon signed ranks tests was used to compare naming accuracy for nouns vs. verbs. Performance on nouns was significantly higher than for verbs for the multiSLI group (*z* = −2.023, *p* = 0.043); noun accuracy was higher than verbs but not significantly so for the multiTLD-LM group (*z* = −1.070, *p* = 0.285). Paired *t*-tests results concurred with the non-parametric ones. The three English-speaking multilingual children were further tested in English and all showed a better performance in their L2 (SMG) compared to their L1b (English), arguably bootstrapped by their close native L1a (CG); noun accuracy was higher than verb accuracy in both languages.

Of the types of errors that were coded, multilingual children with and without SLI made more errors overall than typically developing bilectal children for both nouns and verbs. Omission errors for nouns also appear more frequently in both multilingual groups, where the multiSLI made more verb semantic errors and the multiTLD-LM more verb omission errors. In non-parametric group comparisons on each type of error, it was found that the groups differ significantly on noun and verb omission errors [$\chi_{\left(3\right)}^{2}=$16.615, *p* = 0.001 and $\chi_{\left(3\right)}^{2}=$18.083, *p* < 0.001] as well as verb semantic errors [$\chi_{\left(3\right)}^{2}=$17.948, *p* < 0.001]. Further pairwise comparisons revealed that the two multilingual groups made significantly more omission and verb semantic errors than the typically developing bilectal children. In essence, error type did not distinguish SLI groups (bilectal vs. multilingual).

In sum, multilingual children with SLI, like their monolingual and bilectal language-impaired peers, perform analogously to language-matched children on naming accuracy for verbs and noun on a picture-based naming task. Once more, verbs are significantly more difficult to retrieve than nouns—a finding comparable to the monolingual and bilectal studies conducted so far in the literature (Kambanaros et al., 2013a). Taken together, these data points substantiate the claim that children with SLI, irrespective of whether they are monolingual, bilectal, or multilingual, demonstrate: (i) lexical (word-level) skills similar to younger counterparts with typical language development; (ii) no evidence of deviant or disrupted acquisition in (at least) the lexical domain; (iii) a significantly greater difficulty in retrieving verbs as opposed to nouns; (iv) consistency of omissions as the major error type for nouns across languages; and (v) divergence in the major error type for verbs across languages. This is an issue for the role of language proximity in (impaired) language development, whichever direction it is going to be implemented: Multilingual children do not show different, perhaps “additional,” problems compared to bilingual ones, regardless of the additional language(s)—and not compared to the closely related bilectals either.

Our findings thus constitute the first indication from multilingual children with SLI in support of the *delayed acquisition hypothesis* for SLI (Rice, 2003). The relevance of this becomes obvious once the next step is considered in a language-impaired child's development: appropriate intervention or speech–language therapy. One major issue for speech–language therapists is how to go about treating (multilingual) children with SLI. In a related recent study (Kambanaros et al., 2015), we reported on lexical retrieval deficits using an equivalent-based measure of expressive vocabulary in the three languages of a multilingual school-aged child diagnosed with SLI. In follow-up work (Kambanaros et al., submitted), we carried out a therapy study treating cognates in one of the child's three languages (English) and observed an effect in her other two languages (Bulgarian and Greek).

## Discussion and outlook

Addressing the present *Frontiers* research topic, we take “the grammar of multilingualism” to be a highly complex area of research that by definition needs to include a lot of different measurements—by which we mean, ideally, the investigation of different measures, different sets of data, different populations, all carried out by interdisciplinary research teams. There is a need for thorough sociolinguistic work, putting the languages under investigation into their social and communicative context, for example. There is a need for thorough theoretical linguistic work, identifying the relevant structures and patterns to be investigated. There is a need for thorough psycholinguistic work, designing and carrying out the best possible experimental methodology. There is a need for cognitive psychological work, probing executive control abilities. And there is a need for clinical linguistic work, assessing and treating language impairment.

This list can be added to and enriched in many ways. The bottom line is that the notion of *comparative bilingualism* can be quite useful and instructive for future research activities, especially when carried out across different countries and languages. The narrow goal of this article was thus to draw attention to this state of affairs and elaborate the research path of comparative bilingualism (Grohmann, 2014b), with a focus on Cyprus (Grohmann and Leivada, 2012, 2013; Kambanaros et al., 2013b; Rowe and Grohmann, 2013, 2014; Karpava and Grohmann, 2014). One such intriguing path would be the role of comparative bilingualism for children with developmental language impairment, something we pointed to as well (Kambanaros et al., 2013a, 2014, 2015), even for therapy strategies (Kambanaros et al., submitted).

However, there is also a broader, larger message behind the above. We could only touch on the role of atypical and impaired language development, and only hint at further comparisons with acquired language disorders and language breakdown in age. A particular avenue of research that investigates more closely the commonalities behind these may be couched within what Benítez-Burraco and Boeckx (2014) refer to as *comparative biolinguistics*, that “inter- and intra-species variation that lies well beneath the surface variation that is the bread and butter of comparative linguistics” (Boeckx, 2013, pp. 5–6). This is a larger research enterprise, continuing the list started above. The primary aim is to obtain distinctive linguistic profiles regarding lexical and grammatical abilities, concomitant with the goal to develop cognitive profiles such as executive control across a range of genetically and non-genetically different populations who are bilectal and multilingual, with or without co-morbid linguistic and/or cognitive impairments as part of their genotype. While individual variability is clinically crucial, population-based research can advance cognitive–linguistic theory through behavioral testing that acknowledges the brain bases involved. This will offer a unique opportunity to researchers in cognitive neuroscience, psychology, speech and language therapy/pathology, psycho- and neurolinguistics, and language development to collaborate.

Our more immediate and local hope is to integrate such research backgrounds within CAT, since we believe that Cyprus is predestined to carry out such population-based research rather easily, at least from a logistical perspective: Cyprus is a small country, hosts many different cultural and linguistic backgrounds, has bilectal, bi-, and multilingual speakers, and much of what we report for the Greek-speaking Republic of Cyprus also transfers, almost mirror-like, to the Turkish-speaking occupied northern part of the island; in addition, despite its limited geographical size and population numbers, all relevant and, for clinical linguistic purposes, “interesting” disorders can be found on the island, be it genetic malfunctions, developmental impairments, or acquired disorders. In reality, however, this kind of research could, and should, be picked up anywhere in the world.

For such research, children with developmental language disorders that are language-/behavior-based or as the result of a genetic syndrome should be targeted, including the following pathological conditions which we know exist in Cyprus in research-appropriate numbers:

- Specific Language Impairment (SLI): SLI is considered a language disorder in children exhibiting difficulties acquiring grammar, phonological skills, semantic knowledge, and vocabulary, despite having a non-verbal IQ within the normal range.
- Developmental Dyslexia (DD): Children with DD experience problems learning to read, write, and spell below their chronological age, despite having a non-verbal IQ within the normal range.
- Autism Spectrum Disorders (ASD): Children with a high-functioning ASD, such as Asperger's, have problems with language and communication; they also show repetitive and/or restrictive patterns and thoughts of behavior, despite having a non-verbal IQ within the normal range.
- Down Syndrome (DS): DS is caused by three instead of the normal two copies of chromosome 21; children present with language and cognitive deficits, though differently from WS.
- William's Syndrome (WS): Individuals with WS miss ±28 genes from one copy of chromosome 7; children present with language and cognitive deficits, though differently from DS.
- Fragile X Syndrome (FXS): In FXS a particular piece of genetic code has been multiplied several times on one copy of the X chromosome; children present with language and cognitive deficits.

Each clinical (child) population provides a different perspective on language acquisition and impairment in terms of the relative strengths and weaknesses of certain processes or abilities based on the etiology and are defined as *primary language delay*, where non-linguistic cognitive skills are developing normally (here: SLI and DD), and *secondary language delay*, where language problems are secondary to other conditions (here: ASD, DS, WS, FXS). Statistical procedures can be used to compute and correlate relationships between the research measures and combinations of the background/selection markers. The results will provide new directions for investigating language impairments by considering a broad range of linguistic, cognitive, and behavioral indicators in the realm of bilectalism and multilingualism. This will also allow both associations and dissociations to emerge, and the identification of which factors co-vary with performance scores. In simple terms, it will enable us to understand the “*how*” and the “*why*” of child differences from one to another within and across clinical conditions, and as compared to non-impaired populations.

Putting all of this together, though, there is an even more general issue. Comparing cognitive and linguistic abilities across different populations and different groups of speakers may ask for a further “specialized” area of research. The intention is to compare linguistic and cognitive abilities of monolingual, bidialectal, bilectal, bilingual, and multilingual speakers (comparative bilingualism, with more room for gradience, especially in combination such as Russian–Greek bilinguals in Cyprus) and different language-impaired populations (comparative biolinguistics, unearthing phenotypal variation), who themselves may be on different scales in the gradient spectrum of multilingualism. That is, among the future research participants, there will be vast variation and combinations of “lingual” features, ranging from mono- to multilingualism, from simultaneous to sequential acquisition, from local to heritage language status, from typical development to impairment, from healthy to disorders of various degrees. We tentatively suggest a(nother) new term for this and are excited about what future research may bring: *comparative linguality*.

## Author contributions

Both authors made substantial, direct, and intellectual contribution to the work, and approved it for publication.

### Conflict of interest statement

The authors declare that the research was conducted in the absence of any commercial or financial relationships that could be construed as a potential conflict of interest.
